# Green tea polyphenols alter lipid metabolism in the livers of broiler chickens through increased phosphorylation of AMP-activated protein kinase

**DOI:** 10.1371/journal.pone.0187061

**Published:** 2017-10-26

**Authors:** Jinbao Huang, Yibin Zhou, Bei Wan, Qiushi Wang, Xiaochun Wan

**Affiliations:** 1 State Key Laboratory of Tea Plant Biology and Utilization, School of Tea & Food Science and Technology, Anhui Agricultural University, Hefei, Anhui Province, People's Republic of China; 2 International Joint Research Laboratory of Tea Chemistry and Health Effects, Anhui Agricultural University, Hefei, Anhui Province, People's Republic of China; Rutgers, the State Univesity of New Jersey, UNITED STATES

## Abstract

Our previous results showed that green tea polyphenols (GTPs) significantly altered the expression of lipid-metabolizing genes in the liver of chickens. However, the underlying mechanism was not elucidated. In this study, we further characterized how GTPs influence AMP-activated protein kinase (AMPK) in the regulation of hepatic fat metabolism. Thirty-six male chickens were fed GTPs at a daily dose of 0, 80 or 160 mg/kg of body weight for 4 weeks. The results demonstrated that oral administration of GTPs significantly reduced hepatic lipid content and abdominal fat mass, enhanced the phosphorylation levels of AMPKα and ACACA, and altered the mRNA levels and enzymatic activities of lipid-metabolizing enzymes in the liver. These results suggested that the activation of AMPK is a potential mechanism by which GTPs regulate hepatic lipid metabolism in such a way that lipid synthesis is reduced and fat oxidation is stimulated.

## Introduction

Lipid metabolism in the liver is vitally important for energy homeostasis. Regulation of the expression of genes encoding lipid metabolic enzymes plays a central role in the responses to changes in dietary, nutritional and physiological states. In the treatment of diabetes and obesity with pharmacological agents, the liver is often the target organ[1–3]. Some of these interventional agents, such as metformin, have been shown to increase catabolism in the liver, which yields beneficial effects in diabetic patients.

Some natural products, such as green tea, have also been shown to reduce body weight gain and alter the regulation of glycolipid metabolism of animals *in vivo* [4]. Green tea is one of the most popular beverages in the world. Studies have illustrated that green tea alters fat metabolism [5], likely through the activities of tea polyphenols, among which the catechin (-)-epigallocatechin-3-gallate (EGCG) is the most abundant compound [4]. After the ingestion of tea, EGCG can be detected in the liver[6], indicating that the liver is susceptible to the intervention of dietary catechins. However, the relative importance of this active ingredient under different experimental conditions remains unclear.

Our previous data demonstrated that green tea polyphenols (GTPs) decreased the mRNA level of some lipogenic genes and elevated the expression of several key genes in the fatty acid oxidation pathway in the liver of broiler chickens [7]. However, both the GTP target and the cellular activities GTP influences remained unknown.

A possible target is the AMP-activated protein kinase (AMPK), a vital sensor of energy metabolism at the cellular and whole-organ levels [8]. Activated AMPK can phosphorylate and inhibit acetyl-CoA carboxylase (ACACA), which results in lower malonyl-CoA levels and removal of the inhibition of carnitine palmitoyl transferase 1 (CPT1A) [8]. It has been reported that green tea extract, tea polyphenols, and EGCG could activate hepatic AMPK in rodents and/or cell lines[9–11]. We hypothesized that GTPs would target AMPK in chickens.

To elucidate whether green tea influences the activity or phosphorylation state of AMPK, we examined how GTP treatment of broiler chickens affected AMPK phosphorylation and gene expression of known downstream genes in the liver. Unlike in mammals, the liver of avian species accounts for 95% of *de novo* fatty acid synthesis, making it the most important organ for lipogenesis in the body. Because of this difference in lipid metabolism between birds and mammals, our study provides a unique perspective on the lipid-lowering efficacy of green tea. This data can be further used to develop feed that could result in healthier chickens for human consumption.

## Materials and methods

### Reagents

Green tea polyphenols (GTPs) were purchased from Wuhu Tianyuan Science and Technology Development Co., Ltd. (Anhui, China). The catechin composition (the most important bioactive constituents of green tea) of the GTPs used in this study was assayed by HPLC and found to contain gallic acid (GA, 0.7%), catechin (C, 1.09%), epicatechin (EC, 4.69%), gallocatechin gallate (GCG, 6.23%), epigallocatechin (EGC, 7.63%), epicatechin gallate (ECG, 11.46%), and EGCG (53.1%). The remaining ingredients of the GTP extract (approximately 15%) were anthocyanins, leucoanthocyanins and phenolic acids. Other reagents were either analytical or molecular grade.

### Animal feeding and sampling

Male broiler chicks (Ross 308, *Gallus gallus domesticus*) were obtained from a local hatchery and subsequently raised in a standard chicken facility. The breeding room temperature was maintained at 21°C [45% relative humidity (RH)]. All chickens were provided a grower diet with 19% crude protein and 12.98 MJ/kg metabolizable energy. All birds were given *ad libitum* access to food and water during the entire rearing period.

Animal handling procedures were similar to our previous experiment[7]. All animal experiments were performed in accordance with the ‘‘Guidelines for Experimental Animals” (GB14925-2010, the General Administration of Quality Supervision, Inspection and Quarantine and the Standardization Administration of the People's Republic of China). All animal protocols were reviewed and approved by the Institutional Animal Care and Use Committee (IACUC) of Anhui Agricultural University.

In brief, thirty-six 28-day-old male broilers with comparable body weight were randomly divided into 3 groups (2 chickens per cage; 6 cages per group). After acclimation for 3 days, all animals in each group started to receive one of the following daily oral treatments at 9:00 A.M. for 4 weeks: Control treatment (deionized water); GTP treatment at 80 mg/kg body weight; or GTP at 160 mg/kg body weight. The GTP solutions were freshly prepared in deionized water one hour before oral administration. GTP doses chosen in this research were based on our former results, which is comparable to 0.15% ~ 0.30% dietary GTPs, similar to doses used in rodent studies[12].

On the mornings of days 14 and 28, 6 chickens, one from each cage, were randomly selected from each treatment group. The birds were anesthetized with pentobarbital sodium by intraperitoneal injection. After confirming the effectiveness of the anesthesia by a lack of responsiveness to toe pinching, the chickens were humanely sacrificed by exsanguination. After that, the intraperitoneal cavities were opened and the liver and abdominal fat were harvested, weighed, and washed with ice-cold saline. Liver samples (approximately 100 mg) were placed into 1.5 ml-tubes containing RNAstore reagent (Tiangen, Beijing, China) and stored at -80°C until further analysis of gene expression. The remaining liver samples were immediately frozen in liquid nitrogen and then stored at -80°C.

### Evaluation of hepatic lipid content and fatty acid profile

One gram of liver tissue sample was placed in a 50-ml conical tube, mixed with a chloroform:methanol mixture (2:1, v/v) to a final volume of 20 ml, and homogenized by an IKA T-25 homogenizer in an ice bath for 1 min. The tube was then placed in a shaker at room temperature for 1 h before the protein and connective tissues were removed by vacuum filtration on a Buchner funnel with fat-free filter paper. A solution of physiological saline (20% of the volume of the filtrate) was added and vortexed for several seconds. The tube was centrifuged at 3000 rpm for 15 min. The supernatant was discarded, and the chloroform phase was collected. When needed, the interface of the two phases was washed with a 1:1 water:methanol mixture once or twice. Finally, the chloroform fraction containing the lipid was concentrated by rotary evaporation or by blowing with a stream of nitrogen and precisely weighed to calculate the percent of lipid in the sample (fresh weight). Fatty acid composition was analyzed according to the method of Kandhro et al. [13]. Briefly, the fatty acids were analyzed on an Agilent 6890A gas chromatography instrument (Agilent Technologies, NY). A DB-23 capillary column that was 60 m × 0.25 mm i.d with a 0.25 μm film (Agilent Technologies, Palo Alto, CA) was used for the separation of fatty acid methyl esters. Peak identification was carried out by comparison with the retention times and mass spectra of the known standards. Standard methyl esters of palmitic (76119), stearic (85679), oleic (75090) and linoleic (62230) acids (Sigma-aldrich, CA) were used for confirmation of the mass spectral library (NIST11) identifications.

### Real-time PCR analysis

The expression levels of key genes in the lipid metabolizing pathway were measured using real-time quantitative PCR (RT-qPCR). RNA extraction, reverse transcription and RT-qPCR were the same as our previous study [7]. In brief, total RNA from the liver sample was isolated using a commercial kit (Tiangen, DP431, Beijing, China). Reverse transcription was performed immediately following the total RNA isolation using a first strand cDNA synthesis kit (Takara, 6110A Dalian, China). RT-qPCR was performed using the CFX96^TM^ Real-Time System (Bio-Rad, CA). The expression level of target mRNA was normalized to the mRNA level of beta-actin. The linear amount of the target gene expression to the internal standard was calculated by 2^-△△CT^. The gene sequences involved in the present study were obtained from GenBank (www.ncbi.nlm.nih.gov/Genbank), and the accession numbers and sequences of the primers used are listed in Table 1.

**Table 1:** Gene-specific primers for mRNA amplification of key lipid metabolism genes.

Gene ID	Accession ID	Gene name	primer	Product length
396526	NM_205518	β-actin	Forward: CTGTGCCCATCTATGAAGGCTA	139
			Reverse: ATTTCTCTCTCGGCTGTGGTG	
396061	NM_205155	FASN	Forward: GCAGCTTCGGTGCCTGTGGTT	119
			Reverse: GCTGCTTGGCCCACACCTCC	
396504	NM_205505	ACACA	Forward: AACGAGTCGGGCTACTACCT	119
			Reverse: ATCAGCATCCCGTGAAGTGG	
395706	NM_204890	SCD-1	Forward: CCAGCGGAGATACTACAAGCC	184
			Reverse: CCGATTGCCAAACATGTGAGC	
373915	NM_204126	SREBF-1c	Forward: TCACCGCTTCTTCGTGGAC	220
			Reverse: CTGAAGGTACTCCAACGCATC	
423118	NM_001012898	CPT1A	Forward: TCGTCTTGCCATGACTGGTG	143
			Reverse: GCTGTGGTGTCTGACTCGTT	
417366	NM_001006205	ACOX1	Forward: ATGTCACGTTCACCCCATCC	133
			Reverse: AGGTAGGAGACCATGCCAGT	
374120	NM_001001464	PPARα	Forward: TGTGGAGATCGTCCTGGTCT	103
			Reverse: CGTCAGGATGGTTGGTTTGC	

Notes: β-actin, beta-actin; FASN, fatty acid synthase; ACACA, acetyl-CoA carboxylase; SCD1, stearoyl-coenzyme A desaturase 1; SREBF-1c, sterol regulatory element-binding protein-1c; CPT1A, carnitine palmitoyl transferase 1; ACOX1, acyl-CoA oxidase 1; PPARα, peroxisome proliferator-activated receptor-alpha.

### SDS-PAGE and Western blotting

The liver samples were homogenized in lysis buffer (containing proteinase and phosphatase inhibitors) using a motor-driven pestle in a 1.5-ml conical tube. After being stored on ice for 30 min, the homogenates were centrifuged at 4°C at 12,000 x g for 45 min. The supernatants were harvested, and the protein concentrations were measured. Western blotting followed the procedure of Murase et al. [11]. The bands of the target protein were obtained using a chemiluminescence reagent (Thermo Scientific, MA) and a ChemiDoc XRS imaging system (Bio-Rad, CA).

Primary antibodies for ACACA (Thermo, PA5-17564), phospho-acetyl-CoA carboxylase (Ser79) (Cell Signaling Technologies, #03661, MA), phospho-AMPKα (Thr172) (Millipore, #07–626, MA), and GAPDH (Santa cruz, sc-20357, TX) were used in this experiment. These antibodies were all validated for use with chicken samples according to the instructions of the companies.

### Activity of lipid metabolic enzymes

The activities of hepatic AMPK (GMS50140.2.3), ACACA (GMS50510.2), FASN (GMS50509.2) and CPT1A (GMS50118.2.2) were determined using analytical kits (Genmed Scientifics, MA).

The activity of AMPK was measured by the phosphorylation of a synthetic `SAMS' peptide in a pyruvate kinase—lactate dehydrogenase reaction system. The transformation of reduced nicotinamide adenine dinucleotide (NADH) to nicotinamide adenine dinucleotide (NAD) was measured to determine the amount of ADP produced by the phosphorylation reaction. The pyruvate kinase—lactate dehydrogenase reaction determines the content of ADP. The analysis of ACACA activity was also based on the amount of ADP generated from the ACACA-catalyzed reaction. The CPT1A activity was assayed spectrophotometrically in supernatants of homogenized liver tissues by following the release of CoA-SH from palmitoyl-CoA using the general thiol reagent DTNB (5,5-dithiobis-(2-nitrobenzoic acid)) in Tris-HCl–DTNB buffer (116 mM Tris, 2.5 mM EDTA, 2 mM DTNB, 0.2% Triton X-100, pH 8.0). The activity of FASN was analyzed by the amount of nicotinamide adenine dinucleotide phosphate (NADP^+^) generated from the FASN catalysis reaction using acetyl CoA, malonyl CoA and reduced nicotinamide adenine dinucleotide phosphate (NADPH) as substrates.

### Measurement of the concentration of hepatic malonyl-CoA

The concentration of hepatic malonyl-CoA was measured by an ELISA kit (Shanghai Jianglai Biological Technology Co., Ltd, JL21634, Shanghai, China) according to supplier instructions.

### Statistical analysis

Experimental data were analyzed by the IBM SPSS Statistics 22.0 software (IBM, Armonk, New York, USA). One-way ANOVA and Tukey multiple-range analysis methods were employed to assess the significance of GTP treatments.

## Results

### GTP supplementation on the liver-to-body ratio and abdominal fat mass

During the experimental period, the feed intake and body weight were monitored. Our data indicated that 2 and 4 weeks of GTP treatment did not affect the feed consumption or body weight gain in the experimental birds (Table 2). After 2 weeks of supplementation with GTPs, the liver/body weight ratio and the abdominal fat mass of chickens showed no significant differences among the three groups (Table 2). However, after 4 weeks, the abdominal fat mass of birds fed 80 and 160 mg/kg GTP significantly decreased by 31.82% (*p* < 0.01) and 34.29% (*p* < 0.01), respectively (Table 2). Additionally, the 160 mg/kg GTP group showed a significantly reduced liver/body weight ratio after 4 weeks of treatment (Table 2).

**Table 2:** Effects of green tea polyphenols on feed intake, body weight, liver-to-body weight ratio, and abdominal fat mass in broiler chickens.

	Treatments		
	Control	80 mg/kg GTPs	160 mg/kg GTPs
**Average feed intake** **(g/day/broiler)**^1^			
2-week	137.80±7.67	133.57±9.08	133.27±9.10
4-week	167.03±8.45	164.20±5.09	161.67±9.15
**Body weight** **(kg)**			
2-week	1.66±0.13	1.61±0.13	1.65±0.15
4-week	2.26±0.14	2.25±0.18	2.27±0.22
**Liver/body weight ratio** **(%)**^2^			
2-week	2.07±0.16	2.04±0.20	1.92±0.16
4-week	1.90±0.14	1.87±0.18	1.52±0.14**
**Abdominal fat mass** **(%)**^2^			
2-week	1.73±0.12	1.65±0.14	1.63±0.11
4-week	2.79±0.17	1.90±0.15**	1.83±0.13**

^1^ Average feed intake was calculated by net feed intake (the amount of added feed minus the residual feed every week) divided by 7 days and the number of birds in each cage.

^2^ Liver/body weight ratio and abdominal fat mass were calculated as the percentage of the live body weight of the broiler. The values are means ± SEM; statistical significance was determined by ANOVA with Duncan's multiple-range test

** *p* < 0.01 compared with the control.

### GTP supplementation on the hepatic lipid content and oleic acid proportion

After 2 weeks of treatment with GTPs, no significant differences in liver fat content were observed among the different groups. However, after 4 weeks of treatment, the animals fed GTP showed significantly reduced hepatic lipid levels. The animals supplemented 80 mg/kg and 160 mg/kg GTPs had 7.46% and 8.0%, respectively, lower hepatic fat levels (Table 3). It is worth noting that the changes in liver fat composition appeared to start earlier. After 2 weeks, the ratios of oleic acid to total fatty acids were significantly decreased in broilers fed 80 and 160 mg/kg GTPs (by 24.5% and by 38.5%, respectively; Table 3). A similar reduction in hepatic oleic acid was also observed after 4 weeks of GTP treatment.

**Table 3:** Effects of green tea polyphenols on the fat profile and malonyl-CoA content in the liver of broilers.

	Treatments		
Liver contents	Control	80 mg/kg GTPs	160 mg/kg GTPs
Fat ^1^			
2-week	4.72±0.12	4.77±0.08	4.51±0.10
4-week	5.23±0.09	4.84±0.08**	4.81±0.06**
oleic acid proportion^2^			
2-week	18.17±0.57	13.71±0.58**	11.17±0.84**
4-week	19.50±0.60	15.67±0.92**	10.98±0.35**
malonyl-CoA content (IU/g)			
2-week	8.45±0.30	7.32±0.20	5.97±0.19*
4-week	6.90±0.30	4.56±0.29**	4.17±0.15**

^1^ Fat content was calculated as the percentage of live weight.

^2^ Oleic acid proportion is shown as a percentage of the total fat content of the liver. The values are means ± SEM (n = 6); statistical significance determined by ANOVA with Duncan's multiple-range test, *p < 0.05 and **p < 0.01 compared with the control.

### GTPs altered the expression of lipid-metabolizing genes in the liver

**Fig 1:**
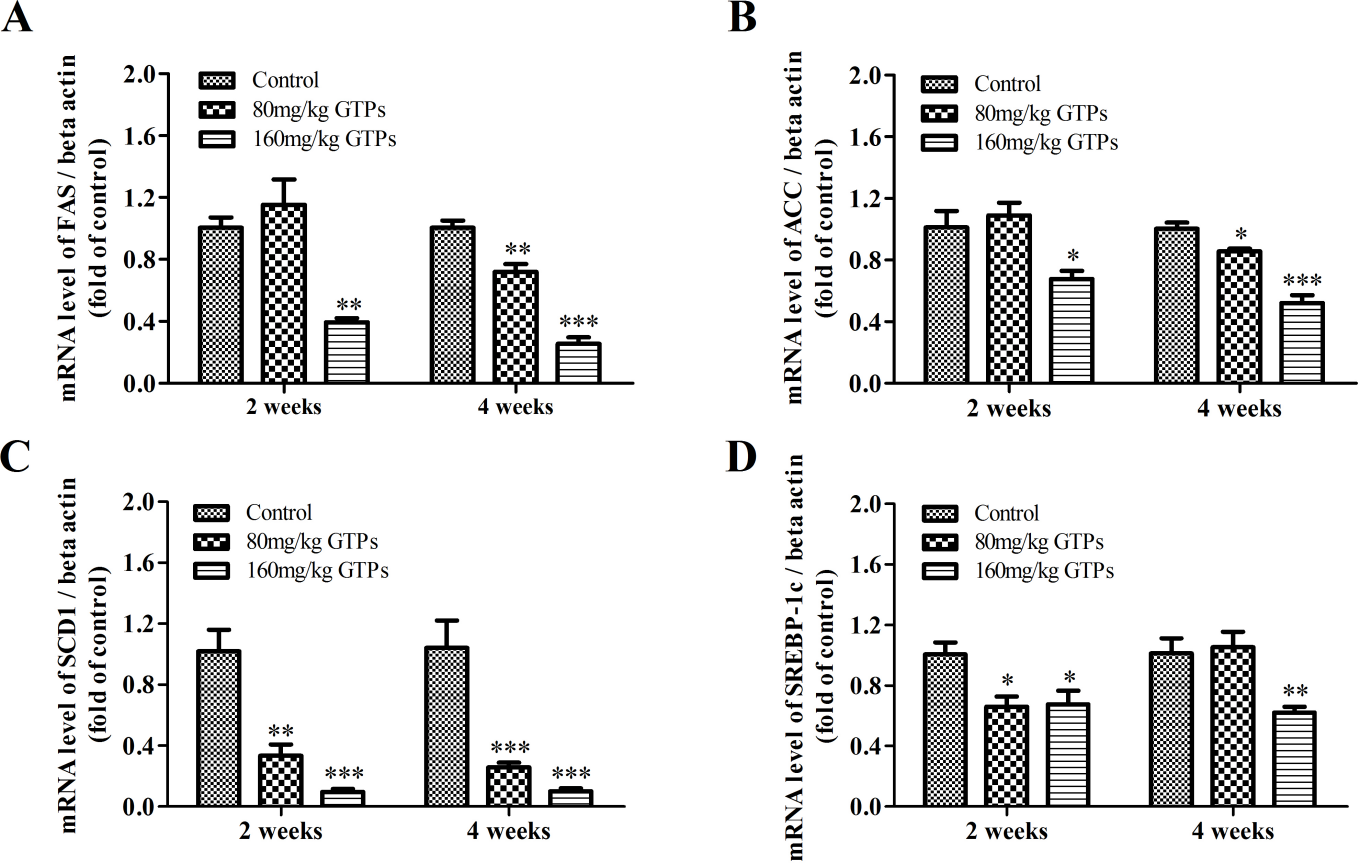
Effects of GTPs on the mRNA expression levels of representative fatty acid synthesis genes in the liver. Broilers were treated with vehicle (distilled water), 80 mg/kg GTPs, or 160 mg/kg GTPs for 2 or 4 weeks: A, fatty acid synthase (FASN); B, acetyl-CoA carboxylase (ACACA); C, stearoyl-coenzyme A desaturase 1 (SCD1); D, Sterol regulatory element-binding protein-1c (SREBP-1c). Values are represented as the means ± SEM (n = 6). Statistical significance was determined by ANOVA with Duncan's multiple-range test, with levels of significance at *p < 0.05, **p < 0.01, and ***p < 0.001 compared with the control.

To elucidate how GTPs modulate lipid metabolism, the gene expression of some key enzymes and factors in the fat metabolizing pathway were determined using RT-qPCR. After 2 weeks of supplementation with 160 mg/kg of GTPs, the expression levels of hepatic FASN and ACACA were significantly decreased, by 61.2% and 33.7%, respectively (Fig 1A and 1B). The mRNA levels of these two lipid synthesis genes were not significantly altered in chickens fed 80 mg/kg GTPs. After 4 weeks of treatment, the expression levels of FASN and ACACA in the livers of broilers fed 80 mg/kg GTPs were reduced by 28.4% and 14.6%, respectively, and in the livers of broilers fed 160 mg/kg by 74.8% and 48.1%, respectively (Fig 1A and 1B). The expression of the desaturase SCD1 was sharply decreased by both doses of GTPs after treatment for 2 or 4 weeks (Fig 1C). The hepatic mRNA levels of SCD1 were significantly lowered after 2 weeks by 68.7% or 92.3%, respectively, with the treatment of 80 or 160 mg/kg of GTPs, and after 4 weeks by 78.4% and 94.1%, respectively, by the low and high doses of GTPs.

Treatment with GTPs also strongly repressed hepatic gene expression of a lipid metabolism-related transcription factor. The gene expression of SREBP-1c in the livers of chickens supplemented 160 mg/kg GTP was reduced by 33.1% or 39.2% after 2 or 4 weeks, respectively (Fig 1D).

**Fig 2:**
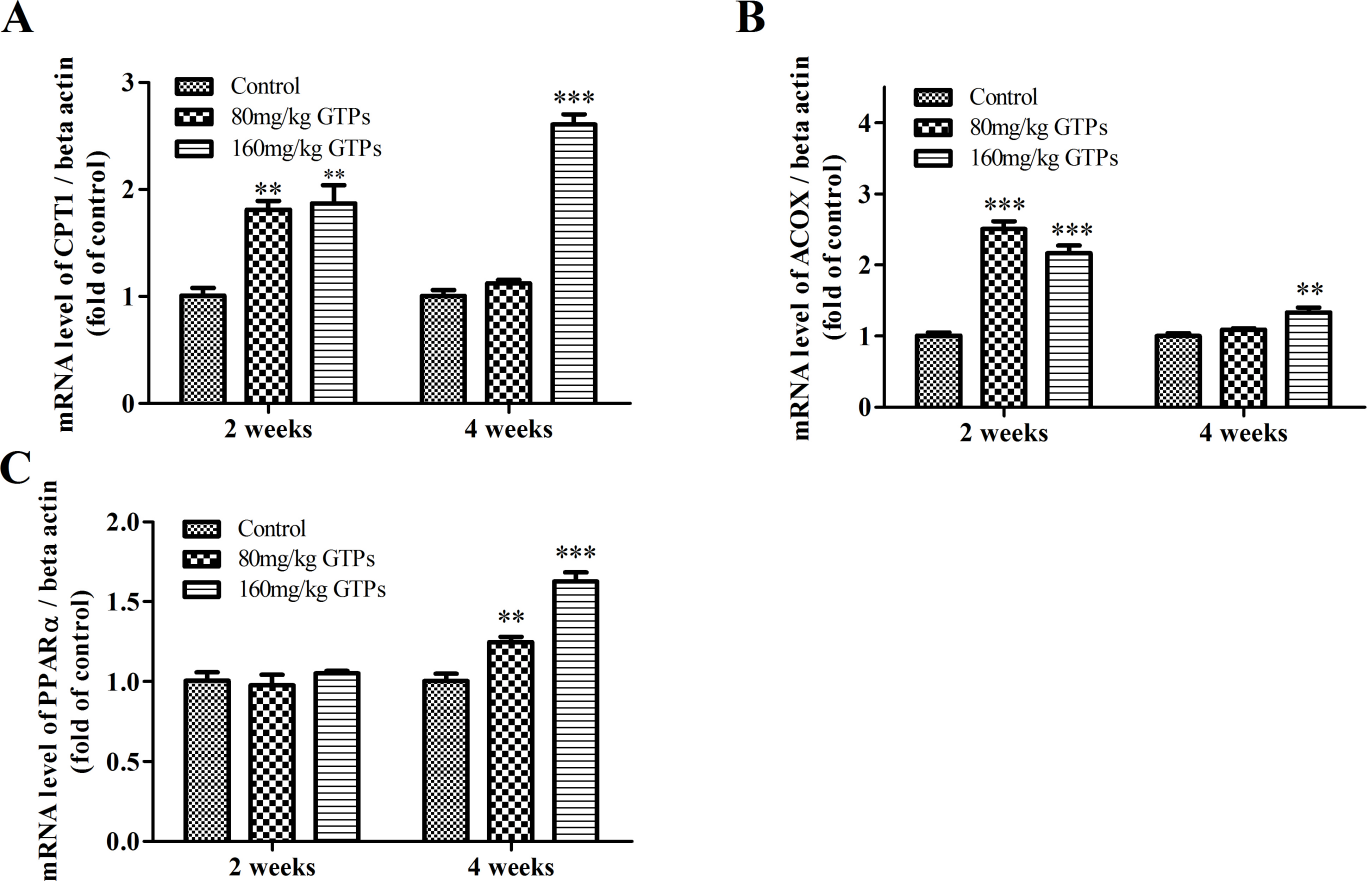
Effects of GTPs on mRNA expression levels of representative lipolysis genes in the liver. Broilers were treated with vehicle (distilled water), 80 mg/kg GTPs, or 160 mg/kg GTPs for 2 or 4 weeks: A, carnitine palmitoyl transferase I (CPT1A); B, acyl-CoA oxidase 1 (ACOX); and C, peroxisome proliferator-activated receptor-alpha (PPARα). Values are represented as the means ± SEM (n = 6). Any statistical significance was determined by ANOVA with Duncan’s multiple-range test, with levels of significance at ** *p* < 0.01 and *** *p* < 0.001 compared with the control.

On the other hand, GTP treatment significantly increased the mRNA expression levels of beta-oxidation genes in the liver (Fig 2). After 2 weeks, the expression level of hepatic CPT1A was significantly enhanced, by 79.3% and 87.4% in the animals from the 80 and 160 mg/kg GTP-treatment groups, respectively (Fig 2A). The gene expression levels of acyl-CoA oxidase 1 (ACOX1) during this period was also significantly increased, by 151.2% and 116.3% in the 80 mg/kg and 160 mg/kg GTP-treatment groups, respectively (Fig 2B).

Similarly, after 4 weeks, a 2.60- and 1.33-fold up-regulation in CPT1A and ACOX1 gene expression, respectively, was observed after treatment with 160 mg/kg GTPs; however, no significant changes were found with the 80 mg/kg GTP treatment at this longer time point. Additionally, the mRNA level of the transcription factor PPARα was significantly enhanced in the liver following 4 weeks of supplementation with 80 mg/kg and 160 mg/kg GTPs (Fig 2C).

### GTPs alter the activity of lipid metabolic enzymes in the liver

**Fig 3:**
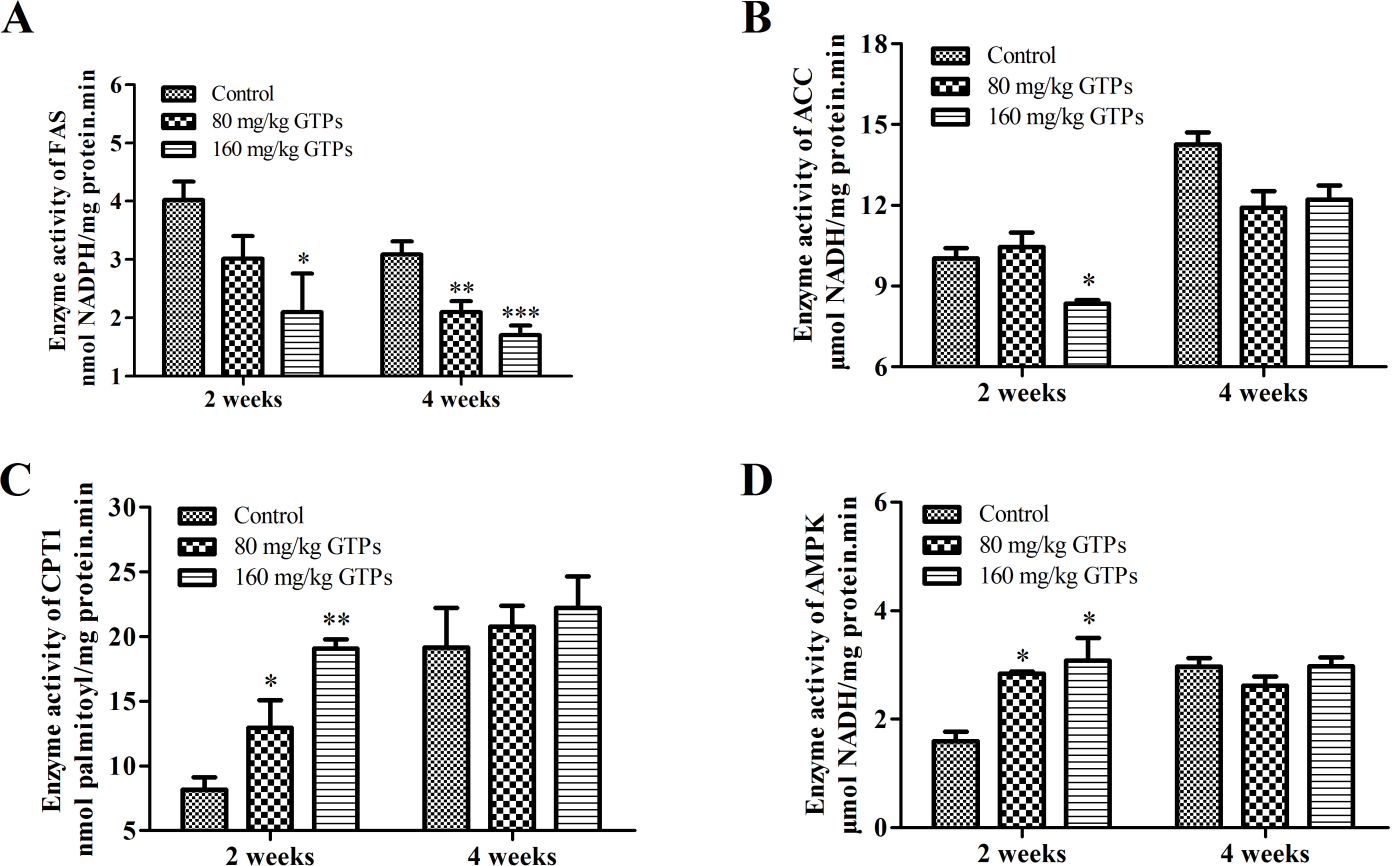
Effects of GTPs on lipid-related enzyme activities in the liver. Broilers were treated with vehicle (distilled water), 80 mg/kg GTPs, or 160 mg/kg GTPs for 2 or 4 weeks: A, fatty acid synthase (FASN); B, acetyl-CoA carboxylase (ACACA); C, carnitine palmitoyl transferase I (CPT1A); and D, AMP-activated protein kinase (AMPK). Values are represented as the means ± SEM (n = 6). Statistical significance was determined by ANOVA with Duncan's multiple-range test, with * *p* < 0.05, ** *p* < 0.01, and *** *p* < 0.001 representing significant differences compared with control.

To further verify the effects of GTPs on fat metabolism in the liver, the activities of some fat metabolizing enzymes were examined. Our data showed that the activities of hepatic ACACA and FASN were significantly decreased, by 16.8% and 47.8%, respectively, after treatment with 160 mg/kg GTPs for 2 weeks (Fig 3A and 3B). After treatment for 4 weeks, the FASN activities were significantly suppressed by 32.1% and 44.7%, in the livers of chickens fed 80 and 160 mg/kg GTPs, respectively. After 2 weeks, the enzymatic activity of CPT1A was significantly increased, by 59.4% and 134.2% in chickens supplemented 80 and 160 mg/kg of GTPs, respectively (Fig 3C). After 4 weeks of treatment, the increase in CPT1A activity was not significant. Also after 2 weeks, the activity of hepatic AMPK was significantly increased, by 78.3% and 93.7% in chickens fed 80 and 160 mg/kg GTPs, respectively (Fig 3D). After 4 weeks, however, no significant difference was observed in the hepatic AMPK activities among the three treatment groups.

### GTPs influence on malonyl-CoA concentration in the liver

To further study the regulatory effects of GTPs on hepatic fatty acid metabolism, we examined the malonyl-CoA content in the liver (Table 3). After 2 weeks of treatment with 160 mg/kg GTPs, the malonyl-CoA content in the liver was significantly reduced, by 29.3%. After 4 weeks of treatment with 80 or 160 mg/kg GTPs, the hepatic malonyl-CoA content was significantly reduced, by 33.8% or 39.5%, respectively.

### GTPs affect the phosphorylation levels of hepatic AMPK and ACACA

**Fig 4:**
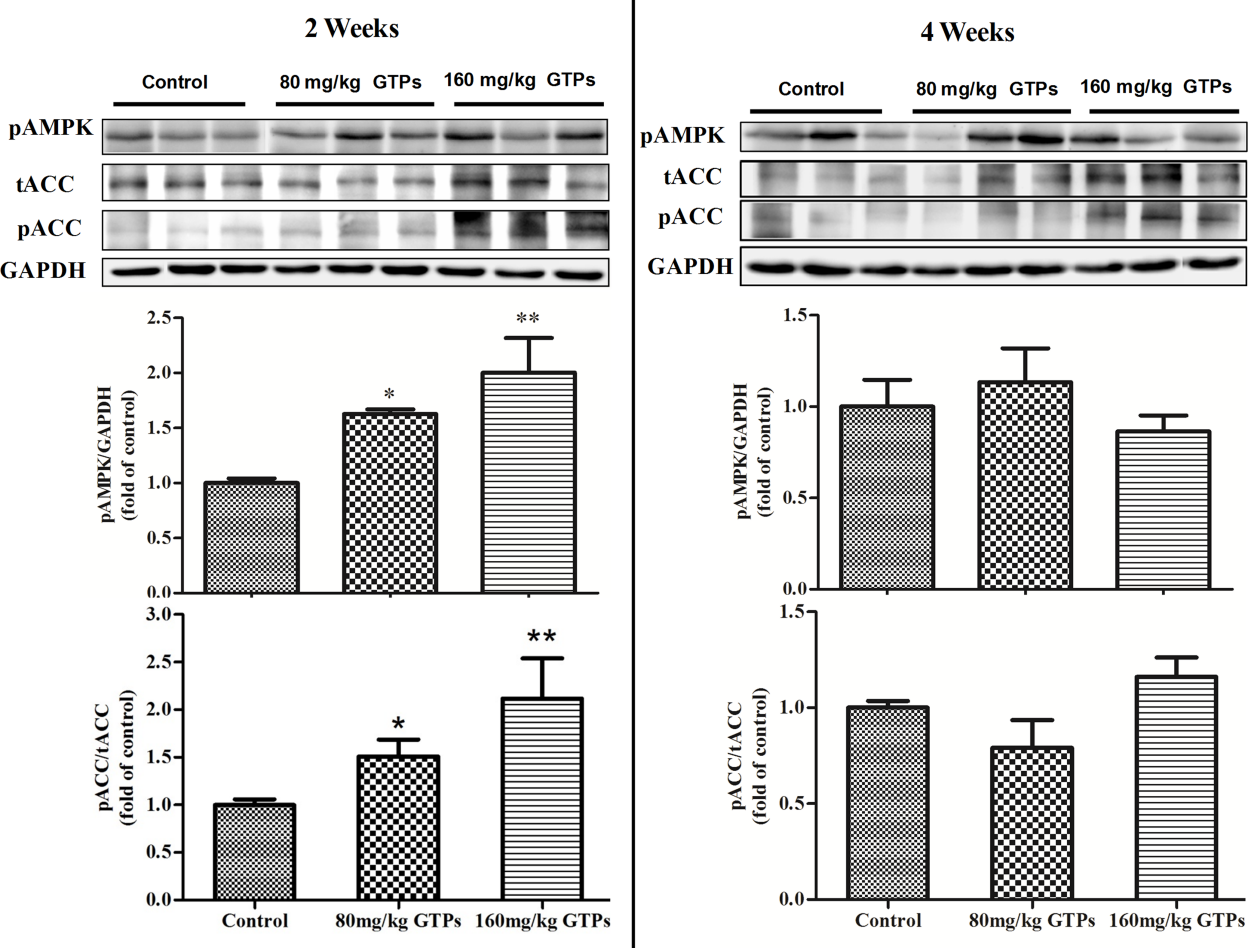
Phosphorylation of hepatic AMPKα and ACACA in broilers treated with green tea polyphenols. Broilers were treated with vehicle (distilled water), 80 mg/kg GTPs, or 160 mg/kg GTPs for 2 or 4 weeks. Western blots were probed with antibodies targeting phosphorylated version of ACACA, acetyl-CoA carboxylase and AMPK, AMP-activated protein kinase or unphosphorylated version of ACACA and GAPDH, glyceraldehyde-3-phosphate dehydrogenase. Values are represented as the means ± SEM (n = 6); statistical significance was determined by ANOVA with Duncan's multiple-range test, with *p < 0.05 and **p < 0.01 compared with control.

To determine if AMPK plays a role in the GTP regulation of lipid-metabolizing genes in the liver, the levels of phosphorylated AMPKα and ACACA in the liver were examined. After 2 weeks of treatment with 80 or 160 mg/kg GTPs, the phospho-AMPKα level was significantly increased, by 63.6% or 101.3%, respectively (Fig 4). However, this effect on phospho-AMPKα was not observed after 4 weeks of treatment (Fig 4). After two or four weeks, the mRNA levels of hepatic AMPKα1 and AMPKα2 were not altered in both GTP treatments (S1 Fig). As a phosphorylation target of AMPK, the level of phospho-ACACA was similarly altered by GTP treatment. A significantly elevated level of phospho-ACACA was observed after 2 weeks of treatment, but not after 4 weeks of treatment (Fig 4).

## Discussion

In animals, lipid synthesis occurs at two major sites: the liver and in adipose tissue. The relative contribution of these sites to lipogenesis varies among species. Differences in the site of fatty acid synthesis and the pattern of lipid trafficking influence not only the overall lipid metabolism in the animal but also the roles of regulatory hormones and transcription factors [14]. For example, hepatic and adipose tissues equally contribute to lipid synthesis in rodents, while in avian species lipogenesis occurs principally in the liver[15]. In this study, we used broiler chickens as a model to investigate metabolic effects of GTPs on lipid synthesis to generate data comparable to that on rodents, in which the benefits of green tea polyphenols on lipid metabolism have been studied extensively.

In this study, GTP supplementation significantly decreased abdominal fat mass and hepatic lipid content in broiler chickens. GTP treatment also altered the mRNA levels of lipid-metabolizing genes in the liver. It is worth mentioning that significant changes in hepatic lipid content were only observed after 4 weeks of treatment. However, most molecular changes in the liver of GTP-treated birds could be detected after 2 weeks, indicating that the expression levels of functional genes were affected before body fat changes were observed. Our data showed that gene expression and enzyme activities of the *de novo* lipid synthesis proteins (FASN and ACACA) in the liver were both suppressed by oral administration of GTPs after 2 and 4 weeks. SCD1 is a key enzyme in monounsaturated fatty acids (Δ^9^) synthesis, which play vital roles in the synthesis of cholesterol esters and triglycerides [16]. In our study, 2 and 4 weeks of treatment with GTPs, especially the higher dose, decreased the gene expression of hepatic SCD1, which was accompanied by a decrease in the ratio of hepatic oleic acid to total lipid content. Our data demonstrated that oral administration of GTPs suppressed fat synthesis in the liver and, considering the role of monounsaturated fatty acids (Δ^9^) in the synthesis of triglycerides and low-density lipoprotein cholesterol, could decrease the lipid content in the extrahepatic tissues of birds.

SREBP-1c is expressed abundantly in the livers of animals and acts as a transcription factor activating lipid synthesis genes such as ACACA, FASN and SCD-1 by binding their promoter regions [17–19]. The reduction of SREBP-1c gene expression in the liver of chickens fed GTP administration is consistent with the decreased hepatic expression of FASN, ACACA and SCD1. In the livers of fructose-fed mice with non-alcoholic fatty acid liver disease, SREBP-1c and its target genes (FASN, ACACA and SCD1) were highly expressed through activation of endoplasmic reticulum (ER) stress in these animals, which subsequently increased lipid synthesis and accumulation in the liver[20]. Recently, it was also suggested that expression of SREBP-1c could be significantly inhibited by leptin[21–23].

In this study, GTP treatment also enhanced the mRNA levels and enzyme activities of lipid-catabolizing genes in the livers. PPARα is a nuclear receptor that is often highly expressed in tissues with a high rate of fatty acid beta-oxidation, such as brown adipose tissue, liver, heart, and muscle, in both avian and mammalian species [24–26]. Its high expression in the liver could enhance the mRNA levels of hepatic CPT1A and ACOX1 [27–29]. These reports are consistent with our results showing that 2 weeks of GTP treatment increased expression of PPARα and the fatty acid beta-oxidation rate-limiting enzymes CPT1A and ACOX1, as well as the increased enzymatic activity of CPT1A.

The present results showing that GTPs suppress the gene expression and enzyme activities of lipid anabolism enzymes and increase the mRNA levels and enzyme activities of lipid catabolism enzymes are consistent with the results from studies with green tea extracts or polyphenols observed in obese mouse and rat models[30–33]. To further characterize the GTP-responsive pathway regulating hepatic lipid metabolism, changes in AMPK were investigated. AMPK is a cellular energy sensor that can switch cellular metabolism from anabolic to catabolic mode[34]. Our data demonstrated that the content of phospho-AMPKα was significantly enhanced in the liver of GTP-treated chickens. Due to the lack of suitable antibodies for AMPKα in chicken, we did not measure the total protein content of AMPKα. However, the data showed that GTP supplementation increased both the activity of hepatic AMPK and the ratio of phosphorylated ACACA and decreased the ACACA activity and the malonyl-CoA level in the liver, suggesting that activating hepatic AMPK is a potential mechanism by which GTPs regulate lipid metabolism in broiler chickens.

In recent years, the effects of tea polyphenols on the AMPK-dependent pathway have been studied using rodents and/or cell lines. For example, green tea extracts (i.g., 50 and 100 mg/kg) in mice have been shown to increase the hepatic phosphorylation of AMPK, as well as its upstream kinase, LKB1, at 3 and 6 h after dosing[9]. The lipid-lowering effect of green tea in high fructose-fed rats was associated with increased AMPK phosphorylation[10]. Orally administered EGCG in mice stimulated energy expenditure, which was also associated with increased phosphorylation of AMPK [11]. AMPK is a short-term sensor of energy metabolism, and an increase in its phosphorylation state can be observed a few hours after administration of green tea polyphenols. In our study, GTP treatment enhanced AMPK phosphorylation at 2 weeks. However, no significant effect of GTPs treatment on phospho-AMPK was observed at 4 weeks. This may be related to an adaptive metabolic response in the liver of birds or an enhanced elimination of catechins during prolonged experimental times. In rats treated with 0.6% GTPs in drinking water, Kim et al. showed that plasma concentrations of catechins reached peak values on day 14 and then slowly decreased to day 1 values on day 28 [35]. Since AMPK is a short-term sensor of energy, the lack of longer-term effects on phosphorylated AMPK may be caused by the decreased level of plasma GTPs in birds. These possibilities remain to be investigated.

### Concluding remarks

Our data demonstrated that the oral administration of green tea polyphenols (GTPs) significantly decreased lipid content, enhanced the levels of phosphorylated AMPKα and ACACA, and altered the mRNA levels and enzymatic activities of lipid-metabolizing enzymes in the livers of broiler chickens. We propose that the activation of AMPK is a potential mechanism through which GTPs alter metabolism, which consequently reduces lipid synthesis and stimulates fat oxidation in the liver.

## Supporting information

S1 Fig Effects of GTPs on mRNA levels of AMPK genes in the liver. Broilers were treated with vehicle (distilled water), 80 mg/kg GTPs, or 160 mg/kg GTPs for 2 or 4 weeks.
(TIF)

S1 Table Raw data for Figs 1–4. Broilers were treated with vehicle (distilled water), 80 mg/kg GTPs, or 160 mg/kg GTPs for 2 or 4 weeks.
(XLSX)

## References

[1] Shaw RJ, Lamia KA, Vasquez D, Koo S-H, Bardeesy N, DePinho RA, et al The kinase LKB1 mediates glucose homeostasis in liver and therapeutic effects of metformin. Science. 2005;310(5754):1642–6. doi: 10.1126/science.1120781 16308421 10.1126/science.1120781 PMC3074427

[2] Foretz M, Hébrard S, Leclerc J, Zarrinpashneh E, Soty M, Mithieux G, et al Metformin inhibits hepatic gluconeogenesis in mice independently of the LKB1/AMPK pathway via a decrease in hepatic energy state. J Clin Invest. 2010;120(7):2355 doi: 10.1172/JCI40671 20577053 10.1172/JCI40671 PMC2898585

[3] Pollak M. Overcoming Drug Development Bottlenecks With Repurposing: Repurposing biguanides to target energy metabolism for cancer treatment. Nat med. 2014;20(6):591–3. doi: 10.1038/nm.3596 24901568 10.1038/nm.3596

[4] Yang CS, Hong J. Prevention of chronic diseases by tea: possible mechanisms and human relevance. Annu Rev Nutr. 2013;33:161–81. doi: 10.1146/annurev-nutr-071811-150717 23642203 10.1146/annurev-nutr-071811-150717

[5] Huang J, Wang Y, Xie Z, Zhou Y, Zhang Y, Wan X. The anti-obesity effects of green tea in human intervention and basic molecular studies. Eur J Clin. Nutr. 2014;68(10):1075–87. doi: 10.1038/ejcn.2014.143 25074392 10.1038/ejcn.2014.143

[6] Suganuma M, Okabe S, Oniyama M, Tada Y, Ito H, Fujiki H. Wide distribution of [3H](-)-epigallocatechin gallate, a cancer preventive tea polyphenol, in mouse tissue. Carcinogenesis. 1998;19(10):1771–6. 9806157 10.1093/carcin/19.10.1771

[7] Huang J, Zhang Y, Zhou Y, Zhang Z, Xie Z, Zhang J, et al Green Tea Polyphenols Alleviate Obesity in Broiler Chickens through the Regulation of Lipid-Metabolism-Related Genes and Transcription Factor Expression. J Agric Food Chem. 2013;61(36):8565–72. doi: 10.1021/jf402004x . 23992224 10.1021/jf402004x

[8] Long YC, Zierath JR. AMP-activated protein kinase signaling in metabolic regulation. Journal of Clinical Investigation. 2006;116(7):1776–83. doi: 10.1172/JCI29044 16823475 10.1172/JCI29044 PMC1483147

[9] Banerjee S, Ghoshal S, Porter TD. Phosphorylation of hepatic AMP-activated protein kinase and liver kinase B1 is increased after a single oral dose of green tea extract to mice. Nutr Res. 2012;32(12):985–90. doi: 10.1016/j.nutres.2012.10.005 . 23244544 10.1016/j.nutres.2012.10.005 PMC3590820

[10] Huang HC, Lin JK. Pu-erh tea, green tea, and black tea suppresses hyperlipidemia, hyperleptinemia and fatty acid synthase through activating AMPK in rats fed a high-fructose diet. Food funct. 2012;3(2):170–7. doi: 10.1039/c1fo10157a 22127373 10.1039/c1fo10157a

[11] Murase T, Misawa K, Haramizu S, Hase T. Catechin-induced activation of the LKB1/AMP-activated protein kinase pathway. Biochem Pharmacol. 2009;78(1):78–84. doi: 10.1016/j.bcp.2009.03.021 . 19447226 10.1016/j.bcp.2009.03.021

[12] Sae-Tan S, Grove KA, Lambert JD. Weight control and prevention of metabolic syndrome by green tea. Pharmacol Res. 2011;64(2):146–54. doi: 10.1016/j.phrs.2010.12.013 21193040 10.1016/j.phrs.2010.12.013 PMC3123415

[13] Kandhro A, Sherazi STH, Mahesar SA, Bhanger MI, Younis Talpur M, Rauf A. GC-MS quantification of fatty acid profile including trans FA in the locally manufactured margarines of Pakistan. Food Chem. 2008;109(1):207–11. doi: 10.1016/j.foodchem.2007.12.029 26054282 10.1016/j.foodchem.2007.12.029

[14] Bergen WG, Mersmann HJ. Comparative aspects of lipid metabolism: impact on contemporary research and use of animal models. J nutr. 2005;135(11):2499–502. 16251600 10.1093/jn/135.11.2499

[15] Gondret F, Ferré P, Dugail I. ADD-1/SREBP-1 is a major determinant of tissue differential lipogenic capacity in mammalian and avian species. J Lipid Res. 2001;42(1):106–13. 11160371

[16] Miyazaki M, Flowers MT, Sampath H, Chu K, Otzelberger C, Liu X, et al Hepatic stearoyl-CoA desaturase-1 deficiency protects mice from carbohydrate-induced adiposity and hepatic steatosis. Cell Metab. 2007;6(6):484–96. doi: 10.1016/j.cmet.2007.10.014 18054317 10.1016/j.cmet.2007.10.014

[17] Kohjima M, Higuchi N, Kato M, Kotoh K, Yoshimoto T, Fujino T, et al SREBP-1c, regulated by the insulin and AMPK signaling pathways, plays a role in nonalcoholic fatty liver disease. Int J Mol Med. 2008;21(4):507–11. 18360697

[18] Améen C, Lindén D, Larsson B-M, Mode A, Holmäng A, Oscarsson J. Effects of gender and GH secretory pattern on sterol regulatory element-binding protein-1c and its target genes in rat liver. Am J Physiol Endocrinol Metab. 2004;287(6):E1039–E48. doi: 10.1152/ajpendo.00059.2004 15280151 10.1152/ajpendo.00059.2004

[19] Higuchi N, Kato M, Shundo Y, Tajiri H, Tanaka M, Yamashita N, et al Liver X receptor in cooperation with SREBP‐1c is a major lipid synthesis regulator in nonalcoholic fatty liver disease. Hepatol Res. 2008;38(11):1122–9. doi: 10.1111/j.1872-034X.2008.00382.x 18684130 10.1111/j.1872-034X.2008.00382.x

[20] Zhang C, Chen X, Zhu R-M, Zhang Y, Yu T, Wang H, et al Endoplasmic reticulum stress is involved in hepatic SREBP-1c activation and lipid accumulation in fructose-fed mice. Toxicol Lett. 2012;212(3):229–40. doi: 10.1016/j.toxlet.2012.06.002 22698815 10.1016/j.toxlet.2012.06.002

[21] Nogalska A, Sucajtys-Szulc E, Swierczynski J. Leptin decreases lipogenic enzyme gene expression through modification of SREBP-1c gene expression in white adipose tissue of aging rats. Metabolism. 2005;54(8):1041–7. doi: 10.1016/j.metabol.2005.03.007 16092054 10.1016/j.metabol.2005.03.007

[22] Yan K, Deng X, Zhai X, Zhou M, Jia X, Luo L. p38 MAPK and LXRα mediate leptin effect on SREBP-1c expression in hepatic stellate cells. Mol Med. 2012;18:10–8. doi: 10.2119/molmed.2011.00243 21979752 10.2119/molmed.2011.00243 PMC3269638

[23] Zhai X, Yan K, Fan J, Niu M, Zhou Q, Zhou Y, et al The β‐catenin pathway contributes to the effects of leptin on SREBP‐1c expression in rat hepatic stellate cells and liver fibrosis. Br J Pharmacol. 2013;169(1):197–212. doi: 10.1111/bph.12114 23347184 10.1111/bph.12114 PMC3632249

[24] Braissant O, Wahli W. Differential expression of peroxisome proliferator-activated receptor-α,-β, and-γ during rat embryonic development. Endocrinology. 1998;139(6):2748–54. doi: 10.1210/endo.139.6.6049 9607781 10.1210/endo.139.6.6049

[25] Diot C, Douaire M. Characterization of a cDNA sequence encoding the peroxisome proliferator activated receptor alpha in the chicken. Poult Sci. 1999;78(8):1198–202. 10472847 10.1093/ps/78.8.1198

[26] Pawlak M, Lefebvre P, Staels B. Molecular mechanism of PPARalpha action and its impact on lipid metabolism, inflammation and fibrosis in non-alcoholic fatty liver disease. J hepatol. 2015;62(3):720–33. doi: 10.1016/j.jhep.2014.10.039 . 25450203 10.1016/j.jhep.2014.10.039

[27] Barger PM, Kelly DP. PPAR signaling in the control of cardiac energy metabolism. Trends Cardiovas Med. 2000;10(6):238–45. doi: 10.1016/S1050-1738(00)00077-3 10.1016/s1050-1738(00)00077-3 11282301

[28] Sa C, Oliveira AR, Machado C, Azevedo M, Pereira-Wilson C. Effects on Liver Lipid Metabolism of the Naturally Occurring Dietary Flavone Luteolin-7-glucoside. Evid Based Complement Alternat Med. 2015;2015:647832 doi: 10.1155/2015/647832 26113868 10.1155/2015/647832 PMC4465769

[29] Ni Q, Shao Y, Wang YZ, Jing YH, Zhang YC. Impact of high altitude on the hepatic fatty acid oxidation and synthesis in rats. Biochem Biophys Res Commun. 2014;446(2):574–9. doi: 10.1016/j.bbrc.2014.03.001 24613835 10.1016/j.bbrc.2014.03.001

[30] Klaus S, Pultz S, Thone-Reineke C, Wolfram S. Epigallocatechin gallate attenuates diet-induced obesity in mice by decreasing energy absorption and increasing fat oxidation. Int J Obes. 2005;29(6):615–23. doi: 10.1038/sj.ijo.0802926 15738931 10.1038/sj.ijo.0802926

[31] Shrestha S, Ehlers SJ, Lee JY, Fernandez ML, Koo SI. Dietary green tea extract lowers plasma and hepatic triglycerides and decreases the expression of sterol regulatory element-binding protein-1c mRNA and its responsive genes in fructose-fed, ovariectomized rats. J Nutr. 2009;139(4):640–5. doi: 10.3945/jn.108.103341 19193814 10.3945/jn.108.103341 PMC2666357

[32] Chen N, Bezzina R, Hinch E, Lewandowski PA, Cameron-Smith D, Mathai ML, et al Green tea, black tea, and epigallocatechin modify body composition, improve glucose tolerance, and differentially alter metabolic gene expression in rats fed a high-fat diet. Nutr Res. 2009;29(11):784–93. doi: 10.1016/j.nutres.2009.10.003 19932867 10.1016/j.nutres.2009.10.003

[33] Lee MS, Kim CT, Kim Y. Green tea (-)-epigallocatechin-3-gallate reduces body weight with regulation of multiple genes expression in adipose tissue of diet-induced obese mice. Ann Nutr Metab. 2009;54(2):151–7. doi: 10.1159/000214834 19390166 10.1159/000214834

[34] Hardie DG. AMP-activated protein kinase: a cellular energy sensor with a key role in metabolic disorders and in cancer. Biochem Soc Trans. 2011;39:1–13. doi: 10.1042/BST0390001 21265739 10.1042/BST0390001

[35] Kim S, Lee M-J, Hong J, Li C, Smith TJ, Yang G-Y, et al Plasma and tissue levels of tea catechins in rats and mice during chronic consumption of green tea polyphenols. Nutr Cancer. 2000;37(1):41–8. doi: 10.1207/S15327914NC3701_5 10965518 10.1207/S15327914NC3701_5

