# Supplementary figures and images for: Green tea polyphenols alter lipid metabolism in the livers of broiler chickens through increased phosphorylation of AMP-activated protein kinase
Source: PLoS One. 2017 Oct 26;12(10):e0187061. doi: 10.1371/journal.pone.0187061 (PMC5658135; doi:10.1371/journal.pone.0187061)

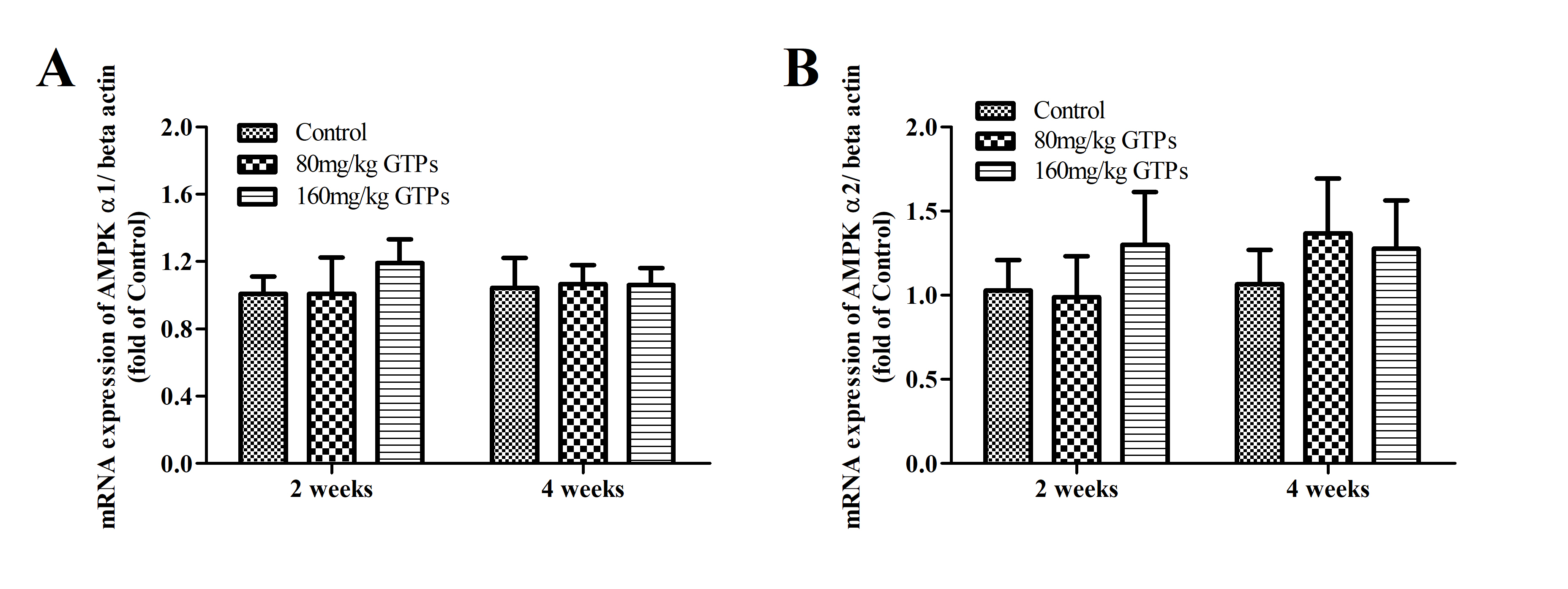

Supplement: S1 Fig — Broilers were treated with vehicle (distilled water), 80 mg/kg GTPs, or 160 mg/kg GTPs for 2 or 4 weeks. (TIF) [file pone.0187061.s001.tif]
